# Implementation of cisternostomy as adjuvant to decompressive craniectomy for the management of severe brain trauma

**DOI:** 10.1007/s00701-020-04222-y

**Published:** 2020-02-03

**Authors:** Lorenzo Giammattei, Daniele Starnoni, Rodolfo Maduri, Adriano Bernini, Samia Abed-Maillard, Alda Rocca, Giulia Cossu, Alexandre Simonin, Philippe Eckert, Jocelyne Bloch, Marc Levivier, Mauro Oddo, Mahmoud Messerer, Roy Thomas Daniel

**Affiliations:** 1 Department of Clinical Neurosciences, Service of Neurosurgery, Lausanne University Hospital (CHUV), Lausanne, Switzerland; 2 Department of Intensive Care Medicine, Lausanne University Hospital (CHUV), Lausanne, Switzerland; 3 University of Lausanne (UniL), Lausanne, Switzerland

**Keywords:** Traumatic brain injury, Decompressive craniectomy, Intracranial hypertension, Cisternostomy

## Abstract

**Objective:**

To evaluate the value of an adjuvant cisternostomy (AC) to decompressive craniectomy (DC) for the management of patients with severe traumatic brain injury (sTBI).

**Methods:**

A single-center retrospective quality control analysis of a consecutive series of sTBI patients surgically treated with AC or DC alone between 2013 and 2018. A subgroup analysis, “primary procedure” and “secondary procedure”, was also performed. We examined the impact of AC vs. DC on clinical outcome, including long-term (6 months) extended Glasgow outcome scale (GOS-E), the duration of postoperative ventilation, and intensive care unit (ICU) stay, mortality, Glasgow coma scale at discharge, and time to cranioplasty. We also evaluated and analyzed the impact of AC vs. DC on post-procedural intracranial pressure (ICP) and brain tissue oxygen (PbO_2_) values as well as the need for additional osmotherapy and CSF drainage.

**Results:**

Forty patients were examined, 22 patients in the DC group, and 18 in the AC group. Compared with DC alone, AC was associated with significant shorter duration of mechanical ventilation and ICU stay, as well as better Glasgow coma scale at discharge. Mortality rate was similar. At 6-month, the proportion of patients with favorable outcome (GOS-E ≥ 5) was higher in patients with AC vs. DC [10/18 patients (61%) vs. 7/20 (35%)]. The outcome difference was particularly relevant when AC was performed as primary procedure (61.5% vs. 18.2%; *p* = 0.04). Patients in the AC group also had significant lower average post-surgical ICP values, higher PbO_2_ values and required less osmotic treatments as compared with those treated with DC alone.

**Conclusion:**

Our preliminary single-center retrospective data indicate that AC may be beneficial for the management of severe TBI and is associated with better clinical outcome. These promising results need further confirmation by larger multicenter clinical studies. The potential benefits of cisternostomy should not encourage its universal implementation across trauma care centers by surgeons that do not have the expertise and instrumentation necessary for cisternal microsurgery. Training in skull base and vascular surgery techniques for trauma care surgeons would avoid the potential complications associated with this delicate procedure.

## Introduction

Severe traumatic brain injury (sTBI) is a life-threatening condition, which continues to cause substantial morbidity and mortality [27]. The pathogenesis of sTBI includes a primary injury, which is directly related to the physical impact onto the brain and a delayed secondary injury which is due to metabolic, excitotoxic, and inflammatory cascades eventually resulting in brain edema, ischemia, and intracranial hypertension [33].

In the setting of sTBI, the development of an uncontrolled intracranial pressure (ICP) is associated with a poor prognosis [22]. Current recommendations have focused on decompressive craniectomy (DC) in sTBI, as a primary procedure (usually after evacuation of a mass effect lesion in case of brain swelling) or as a secondary procedure, in cases of refractory ICP despite maximal medical therapy [33]. DC proved to be effective in reducing ICP and mortality [33], but its effects on outcome are still under debate [10, 16, 17].

Cisternostomy has been recently proposed in the setting of severe TBI as an adjuvant surgical technique that may have a potential for effectively improving ICP control and outcomes [7, 12]. The procedure consists of the opening of the cisternal spaces and draining this compartment for a period of approximately 1 week. The rationale of the procedure lies in the recognition of the important contribution of the paravascular Virchow-Robin spaces to CSF circulation. Subarachnoid hemorrhage, which is almost always present in sTBI, increases the intracisternal pressure that provokes a shift of fluid from the cisternal compartment to the brain parenchyma (“CSF shift edema”). [9] In this situation, cisternostomy may be useful in reversing this fluid shift, thus alleviating brain edema and thereby lowering ICP. We added cisternostomy to our Institutional protocol for the treatment of traumatic brain injury in 2017. According to modified institutional treatment protocol, cisternostomy is performed as a complementary measure to the decompressive craniectomy both in the setting of primary and secondary DC. The aim of this study was to evaluate the contribution of cisternal drainage in the surgical treatment of sTBI.

## Methods

### Patients

We performed a single-center retrospective quality control analysis of a consecutive series of adult patients (≥ 18 years old) who were admitted at the University Hospital of Lausanne with sTBI (Glasgow coma scale ≤ 8 after resuscitation) and underwent surgical treatment, between 2013 and 2018. Cisternostomy was added onto our institutional protocol in January 2017 as an adjuvant surgical procedure to DC. Patients were all managed according to a written algorithm for sTBI and in line with standardized international guidelines [6]. In this written management algorithm, criteria for surgical procedure following sTBI are described as follow:

- Primary surgical procedure: Patients presenting a concomitant predominantly unilateral mass effect such as an acute subdural hematoma greater than 10 mm or a midline shift greater than 5 mm on computed tomographic (CT).
- Secondary surgical procedure: All patients who had refractory ICP despite medical management (based on intracranial pressure monitoring).

The following conditions precluded surgical treatment:

- Brainstem dysfunction and signs of irreversible brain damage (i.e., bilaterally non-reactive pupils)
- Severe hemodynamic instability (i.e., polytrauma)
- Hemorrhagic diathesis

The study had approval from the local ethical Committee (CER-VD, protocol number 2019-00577). Waiver of consent was granted because the procedure was part of our written algorithm for the management of sTBI.

#### Intracranial monitoring

A bolt was placed in the frontal lobe of the most damaged hemisphere including ICP (Codman, Raynham, Massachusetts, USA) and PbtO_2_ probes. (Licox, Integra Neurosciences, Plainsboro, New Jersey, USA). For patients undergoing open cranial surgery for a mass effect lesion, the same probes were placed ipsilaterally and subcutaneously tunneled. Correct placement of all monitors was verified within 24 h by a non-contrast head CT scan.

#### General management of sTBI

Patients were treated according to a standard protocol for the management of severe TBI, in line with the current recommended guidelines [6]. All patients were sedated and mechanically ventilated, aiming to keep PaO2 and PaCO2 between 90 and 100 mmHg and 36 and 40 mmHg, respectively. Cerebral perfusion pressure was maintained between 60 and 70 mmHg, with the use of isotonic fluids and vasopressors. Metabolic control included the maintenance of normoglycemia and normothermia.

#### Management of intracranial hypertension

Elevated ICP was managed sequentially with elevation of head level, deep sedation, analgesia, and muscle paralytics. Optimized moderate hyperventilation (PaCO_2_ 30–35 mmHg) and hypothermia were employed as a second step. If ICP remained superior to 25 mmHg, osmotherapy consisting of intravenous bolus (over 20 min) of 7.5% hypertonic saline (2 mL/kg) or 20% mannitol (0.5 g/kg) was administered. Secondary surgical treatment was considered if the medical therapy failed to keep ICP below 25 mmHg for more than 1 h. Barbiturate coma was not part of the standard management algorithm. ICP control was coupled with PbtO_2_ optimization, which included aggressive management of elevated ICP, plus a sequential stepwise management with MAP/CPP augmentation by way of vasopressors (norepinephrine) and optimization of systemic oxygenation/ventilatory parameters, according to our previously described algorithm [3]. Medical management protocol was the same regardless the surgical strategy adopted.

#### Surgical treatment

The surgery consisted of a decompressive craniectomy (DC) for all patients and the evacuation of any significant hematomas (subdural or parenchymal) if present. The DC for all patients, in this series, included a craniectomy with a medial margin approximatively 1 cm from the midline, an antero-posterior diameter of at least 12 cm and inferiorly reaching the middle cranial fossa floor. The durotomy was made in a stellate fashion which was then augmented with an expansive duraplasty. Starting on January 2017, at our center, we introduced the procedure of an adjuvant cisternostomy (AC) for patients that fulfilled the same inclusion/exclusion criteria as those in whom DC alone was performed. This procedure, which was previously described [7, 14], consisted in opening the basal cisterns to atmospheric pressure and placing a catheter within the cisternal compartment. The head, fixed in a Mayfield clamp, is rotated approximatively 30° to the contralateral side and extended. The skin incision and craniotomy is similar to a classical “trauma flap”. The craniotomy is extended towards the skull base by the epidural drilling of the sphenoid ridge, ideally up to the superior orbital fissure. A frontotemporal durotomy is performed in a curvilinear fashion close to the basal dura, to avoid precocious brain herniation through the durotomy. A lateral subfrontal approach allows an early access to the opticocarotid cistern. The opening of this cistern followed by the adjoining cisternal spaces allows a progressive and dramatic relaxation of the brain. This enables the subsequent opening of the membrane of Liliequist and lamina terminalis. A standard ventricular drain is placed in the cisternal compartment and then subcutaneously tunneled and secured to the scalp. The bone flap is not replaced.

The amount of epidural drilling should be tailored based on the radiological findings. In cases of a large subdural hematoma, there is usually no need for extended epidural drilling because the evacuation of the hematoma in in the frontal and temporal basal regions allows access to the opticocarotid cistern. When the brain edema is severe (especially secondary surgical procedure for refractory ICP), the surgery can be challenging. In these cases, the surgery is quite similar to that of an aneurysmal SAH (aSAH) in the acute phase. It requires increased epidural basal drilling to allow an early access to the cisterns though the narrow subfrontal corridor. The opening of opticocarotid cistern then makes the subsequent cisternal openings easier to accomplish the rest of the procedure as described.

AC was added onto the institutional sTBI surgery protocol in 2017 based on an internal multidisciplinary review process, where it was decided that this procedure would only be performed by surgeons who were experienced in operating on acute aneurysms on a regular basis. The decision of the nature of the surgery (AC vs DC) was dependant on the availability of vascular surgery expertise and was not dependent on the GCS, pupillary dilatation or presence/absence of large mass lesions. The patients in this study were treated by multiple surgeons all experienced in surgery for trauma and were board certified. The only difference with the surgeons who performed AC was that they had an additional expertise in acute aneurysm surgery.

#### Data collection

The clinical data extracted from the records included the patient demographic data, GCS at presentation, pupillary size/reactivity, associated injuries and duration of surgery. The early clinical outcome measures studied were the duration of postoperative ventilation, duration of intensive care unit (ICU) stay, GCS at discharge from ICU, early mortality (during ICU stay) and time to cranioplasty. The details of osmotherapy and amount of CSF drainage were calculated. The long-term clinical outcome (6 months after surgery) was assessed by using the extended Glasgow outcome scale (GOS-E) which was dichotomized as “favorable” (GOS-E was ≥ 5) and “unfavorable” (GOS-E < 5).

The analyzed radiological features at admission assessed on a computed tomography (CT) were the following: (1) midline shift in millimeters, (2) presence and size of a mass lesion such as an acute subdural hematoma (aSDH), epidural hematoma (EDH) and an intracerebral hematoma (ICH), (3) global severity of the brain injury based on the Rotterdam CT score [23]. Complications like neurovascular injuries, iatrogenic contusions, postsurgical hematomas, contusion blossoming, and hydrocephalus (necessitating CSF shunt placement) were noted. Outward brain herniation through craniectomy defect was calculated on postoperative CT scan performed between the 3rd and 5th postoperative according to the criteria presented by Bruno et al. and expressed in centimeters (cm) [4]. Neuromonitoring data were analyzed for each patient, by calculating the mean values of ICP and mean PbO_2_ that were collected hourly for 3 days after surgery (72 h) and also for 6 h before surgery in the secondary surgical procedure subgroup.

#### Statistical analysis

Univariate comparisons between the two groups (DC vs. AC) were performed with a *t* test study according to the underlying distribution for the continuous variables. For categorical variables, Fisher’s exact test was performed. Significance was assessed at *p* < 0.05. All analyses were performed using the statistical software package STATA version 15 (College Station, TX, StataCorp LP).

## Results

**Fig. 1:**
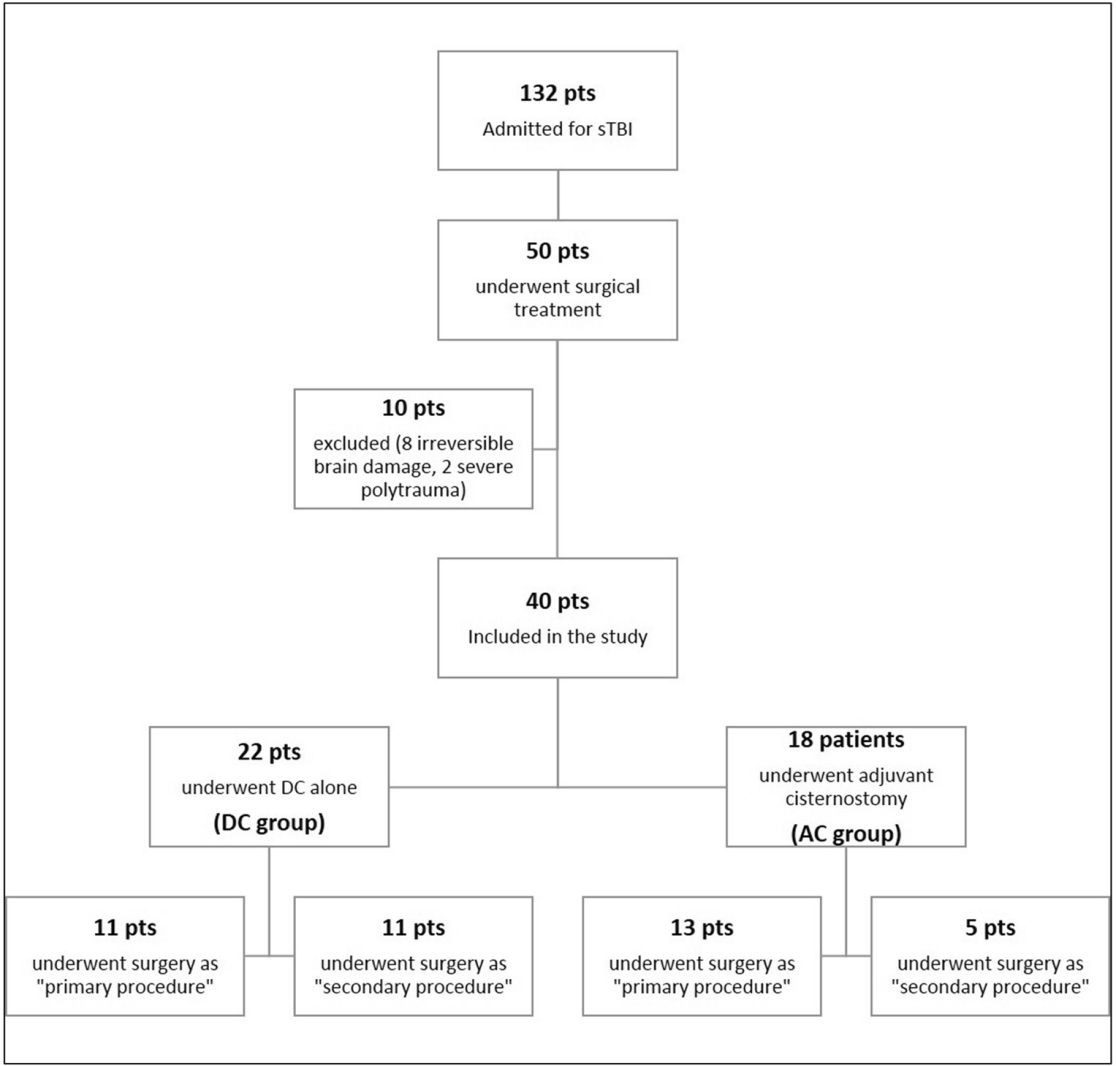
Flow chart showing the treatment pathway for patients admitted to our center with sTBI

From the University Hospital of Lausanne TBI database, we identified 132 patients admitted for sTBI between 2013 and 2018. A total of 50 patients underwent surgical treatment, of which ten patients were excluded based on aforementioned exclusion criteria (Fig. 1). The remaining 40 patients were studied as two distinctive groups (based on the surgery performed) for the subsequent comparative analysis. Twenty-two patients were included in the DC group and 18 in the AC group (Table 1 and Fig. 1). The two groups (AC vs DC) were homogeneous with respect to age (*p* = 0.8) and GCS at presentation (*p* = 0.8); the AC group presented a higher percentage of unilateral mydriasis (55% vs 36%). Concerning the radiological characteristics the AC group presented a significant higher Rotterdam CT score 4.7 vs 3.8 (*p* = 0.03) but were comparable with respect to midline shift. In the primary procedure subgroup, all but one patient had an acute SDH. The AC and DC groups were comparable with respect to type and size of mass lesions (Table 2).

**Table 1:** Clinical, radiological, and outcome characteristics

	DC (22 pts)	AC (18 pts)	
Preoperative characteristics			
Mean age (SD)	48.4 ± 20.4	49.9 ± 19	*P* = 0.8
Male/female	18/4	12/6	
Mean GCS at admission	5.7	5.8	*P* = 0.8
Unilateral pupillary dilation	8 (36%)	10 (55%)	
Primary surgical procedure	11 (50%)	13 (72%)	
Length of surgery in min (SD)	178 ± 30	204 ± 43	*P* = 0.09
Mean Rotterdam Score	3.8	4.7	*P* = 0.03*
Midline shift in mm (SD)	7.1 (6.3)	10.8 (6.4)	*P* = 0.07
Postoperative characteristics			
Days on ventilation (SD)	12.9 ± 6.7	8.5 ± 5.6	*P* = 0.03*
ICU stay (SD)	16.9 ± 7.6	11.9 ± 7.3	*P* = 0.04*
No. of patients requiring osmotherapy	11 (50%)	3 (16.7%)	
Boli of osmotherapy per patient	2.8	0.2	*P* = 0.02*
Early mortality	6 (27.3%)	4 (22%)	
Mean GCS at discharge	10.9	13.1	*P* = 0.001*
6-month FU mean GOS-E (SD)	3.6 ± 2.1	4.8 ± 2.5	*P* = 0.1
6-month FU GOS-E ≥ 5	7/20^†^ (35%)	11/18 (61%)	*P* = 0.1
Mean brain outward herniation in cm	0.86 ± 0.67	0.37 ± 0.85	*P* = 0.05
Time to cranioplasty in days	55.6 ± 40.8	32.5 ± 20.9	*P* = 0.1

*DC* Decompressive craniectomy group; *AC* Adjuvant cisternostomy group; *SD* Standard deviation; *pts.* Patients; *ICU* Intensive care unit; *No.* Number; *GCS* Glasgow coma scale; *FU* Follow-up; *GOS-E* Extended Glasgow outcome scale

*Statistically significant

^†^two patients lost at follow-up

**Table 2:** Clinical, radiological, and outcome characteristics of the primary procedure subgroup

	DC (11 pts)	AC (13 pts)	
Preoperative characteristics			
Mean age (SD)	60.1 ± 12.5	55.6 ± 18.5	*P* = 0.5
Mean GCS at admission	5	5.6	*P* = 0.4
Unilateral pupillary dilation	6 (54.5%)	9 (69.2%)	*P* = 0.6
Mean Rotterdam score	4.8	5.1	*P* = 0.4
Mean midline shift in mm (SD)	11.3 (6.3)	13.5 (5.4)	*P* = 0.4
Hemorrhagic lesion			
EDH	1 (9%)	0 (0%)	*P* = 1
SDH	11 (100%)	12 (92.3%)	
ICH	4 (36.4%)	2 (15.3%)	
Mean size of SDH in mm (SD)	11.7 (7.8)	15.9 (6.1)	*P* = 0.2
Mean volume of ICH in cm^3^ (SD)	29.5 (13.5)	35.4 (5.2)	*P* = 0.6
Postoperative characteristics			
Days on ventilation (SD)	14.4 ± 6.3	7.7 ± 5.5	*P* = 0.01*
ICU stay in days (SD)	18.4 ± 6.4	10.9 ± 7.3	*P* = 0.01*
Mean GCS at discharge	10.4	13.5	*P* = 0.2
6-month FU GOS-E ≥ 5	2/11 (18.2%)	8/13 (61.5%)	*P* = 0.04*
Mean brain outward herniation in cm (SD)	0.63 ± 0.6	0.2 ± 0.9	*P* = 0.2
Mean time to cranioplasty in days (SD)	55.6 ± 40.8	32.5 ± 20.9	*P* = 0.1

*DC* Decompressive craniectomy group; *AC* Adjuvant cisternostomy group; *SD* Standard deviation; *pts.* Patients; *SDH* subdural hematoma; *EDH* epidural hematoma; *ICH* intracerebral hemorrhage; *ICU* Intensive care unit; *No* Number; *GCS* Glasgow coma scale; *FU* Follow up; *GOS-E* Extended Glasgow outcome scale

*Statistically significant

The surgical procedure was slightly longer in the AC group (204 ± 43 min on average) compared with the DC group (178 ± 30.5 min) (*p* = 0.09). The AC group showed a significant shorter duration of mechanical ventilation (*p* = 0.04), shorter ICU stay (*p* = 0.04), and better GCS at discharge (*p* = 0.001) with a similar early mortality in both groups (22% vs. 27%).

In the DC group, one patient (4.5%) showed dramatic enlargement of a hemorrhagic contusion associated with elevated ICP the day after DC, necessitating a surgical evacuation of the mass lesion. One patient (4.5%) showed contusion blossoming without mass effect, therefore not necessitating further surgical procedures. One patient (4.5%) needed an EVD placement 3 days after DC due to unsatisfying ICP control.

In the AC group, cisternostomy was successfully performed in all cases. The lateral sub-frontal access to the cisterns was safely done in all cases. In case of a tight brain, an increased epidural basal drilling was performed that allowed adequate cisternal access, an immediate brain relaxation and placement of the cisternal drain. No neurovascular or frontal lobe damage occurred in relation to the cisternal access. Two patients developed a subcutaneous hematoma necessitating surgical evacuation, the day following surgery. Cisternal CSF drainage was maintained for a mean of 7.2 days [SD 3.3] with a mean CSF drainage in the first 72 postoperative hours of 207 mL/day. Dysfunction of CSF drainage system was not observed in any patient except for one case where there was an inadvertent drain pull out.

**Fig. 2:**
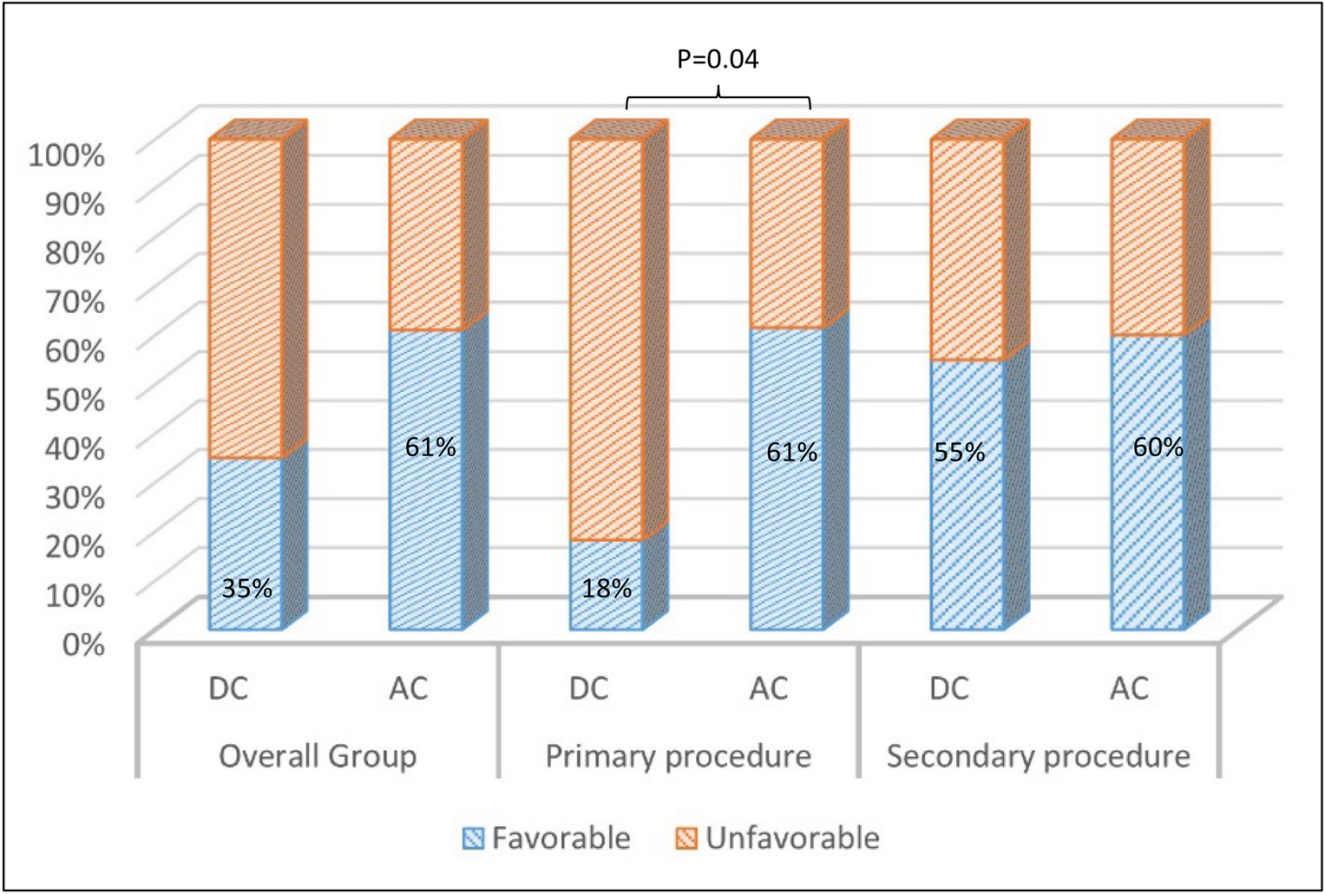
Comparative histograms showing the clinical outcome, dichotomized as favorable (GOS-E ≥ 5) and unfavorable (< 5) in the overall population and subgroups

Mean time to cranioplasty was inferior in the AC group (32.5 vs 55.6 days) though without reaching a statistical difference (*p* = 0.1). Mean brain outward herniation was 0.37 ± 0.87 cm in the AC group and 0.86 ± 0.67 cm in the DC group (*p* = 0.05). At 6-month follow-up (FU), the clinical outcome was considered as favorable (GOS-E was ≥ 5) in 61.1% of patients in the AC group and in 35% of patients in the DC group (*p* = 0.1) (two patients in the DC were lost at FU) (Table 1 and Fig. 2).

### Subgroup analysis (primary vs secondary procedure)

In 24 cases (60%), surgery was performed as “primary”, while in the other 16 patients (40%), a “secondary” procedure was performed on an average of 4 (1–10) days after trauma (Tables 2 and 3 and Fig. 1).

**Table 3:** Clinical, radiological and outcome characteristics of the secondary procedure subgroup

Preoperative characteristics			
	DC (11 pts)	AC (5 pts)	
Mean age (SD)	36.5	34.8 ±10.7	*P*=0.5
Mean GCS at admission	6.3	6.4	*P*=0.9
Unilateral pupillary dilation	2 (18%)	1 (20%)	*P*=1
Mean Rotterdam score	2.9	3.6	*P*=0.005*
Mean midline shift in mm (SD)	2.9 (2.5)	3.8 (2.1)	*P*=0.5
Postoperative characteristics			
	DC (11 pts)	AC (5 pts)	
Days on ventilation (SD)	11.2 ± 6.9	10.8 ± 5.8	*P*=0.9
ICU stay in days (SD)	15.2 ± 8.9	14.4 ± 7.4	*P*=0.8
Mean GCS at discharge	11.5	12.4	*P*=0.3
6-month FU GOS-E ≥5	5/9^†^ (55.6%)	3/5 (60%)	*P*=1
Mean brain outward herniation in cm (SD)	1.1 ± 0.71	0.6 ± 0.56	*P*=0.2
Mean time to cranioplasty in days (SD)	75 (54.6)	50 (22.6)	*P*=0.4

*DC* Decompressive craniectomy group; *AC* Adjuvant cisternostomy group; *SD* Standard deviation; *pts* Patients; *SDH* subdural hematoma; *ICU* Intensive care unit; *No* Number; *GCS* Glasgow coma scale; *FU* Follow up; *GOS-E* Extended Glasgow outcome scale

*Statistically significant

^†^Two patients lost at follow-up

Patients with AC in the “primary procedure” subgroup had a significant shorter ventilation time (*p* = 0.01), shorter ICU stay (*p* = 0.01), a better GCS at discharge (*p* = 0.002) and a better clinical outcome at 6 months (*p* = 0.04) (Table 2 and Fig. 2). In the “secondary procedure” subgroup, a favorable outcome at 6 months was obtained in 60% of the AC group compared with 55.6% of patients in the DC group, and these results did not reach statistical significance due to the low numbers in the subgroup analysis (Table 3 and Fig. 2).

#### Comparative neuromonitoring analysis

**Fig. 3:**
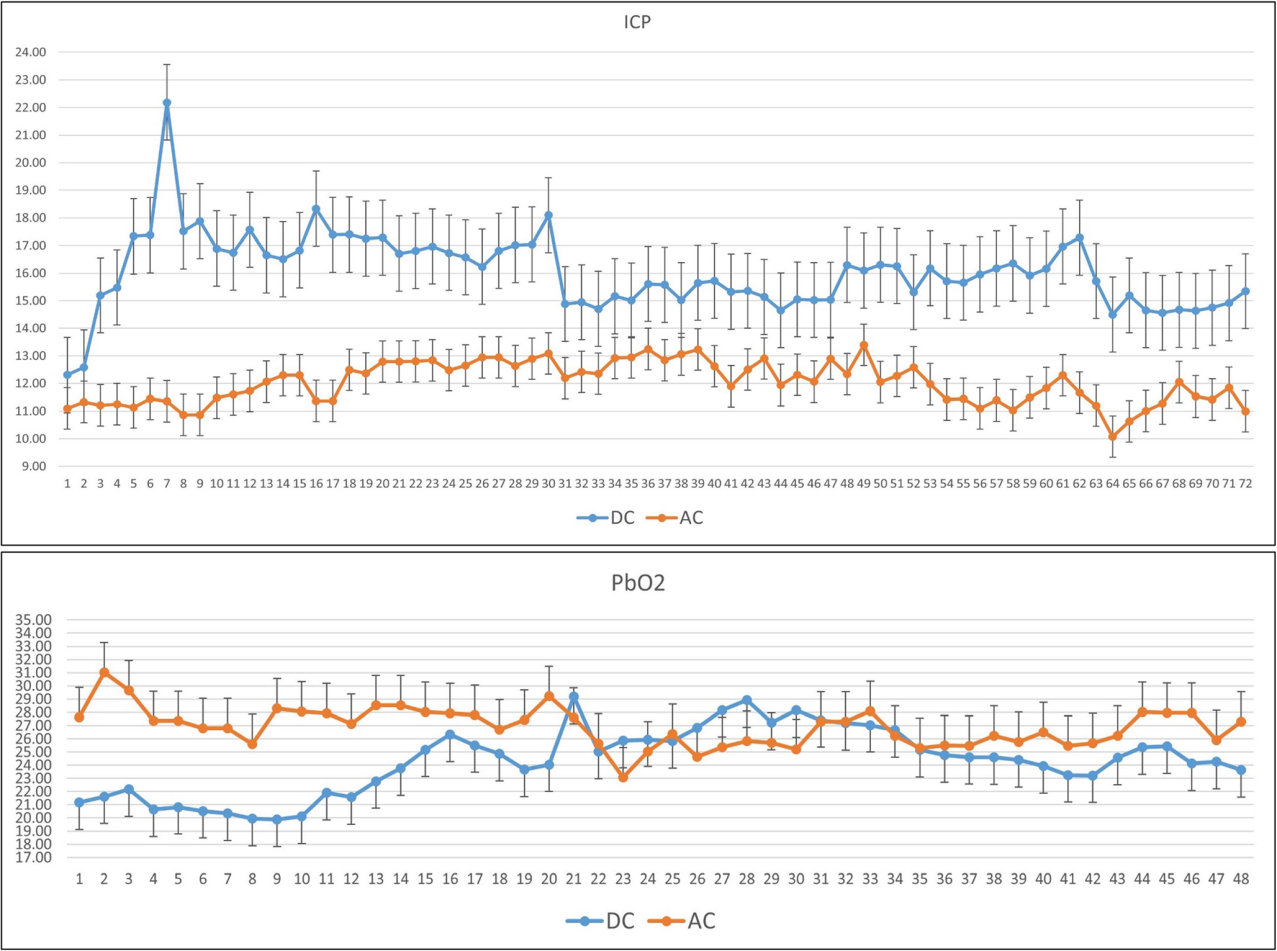
Neuromonitoring curves (ICP and PbO2) showing the mean hourly values after surgery in the overall group

In the overall analyzed period (72 h after surgery), the patients in the AC group presented lower mean ICP values, 12 mmHg (SD 0.8) vs 16 mmHg (1.4) (*p* = 0.0001) compared with DC group, and this difference remained stable overtime. Moreover, in the AC group the hourly averages were always below the threshold of 15 mmHg (Table 4 and Fig. 3). Mean PbO2 showed the same trend with significant higher values in the AC group (*p* = 0.0004), especially in the first 24 h after surgery (*p* = 0.00001).

**Table 4:** Neuromonitoring data

Overall population			
	DC	AC	
Mean post-op ICP (SD)	16 (1.4)	12 (0.8)	*P* < 0.00001
Mean post-op ICP first 24 h (SD)	16.8 (1.8)	11.8 (0.7)	*P* < 0.00001
Mean post-op PbO2 (SD)	24.3 (2)	25.7 (2.3)	*P* < 0.0004
Mean post-op PbO2 first 24 h (SD)	23 (2.5)	27.4 (1.6)	*P* < 0.00001
“Primary procedure” subgroups			
	DC	AC	
Mean post-op ICP (SD)	13.6 (1.9)	11.1 (0.8)	*P* < 0.00001
Mean post-op PbO2 (SD)	22.9 (2.6)	22.2 (2.6)	*P* = 0.1
“Secondary procedure” subgroups			
	DC	AC	
Mean pre-op ICP (SD)	21.3 (6.9)	20.3 (1.5)	*P* = 0.7
Mean pre-op PbO2 (SD)	26.3 (0.7)	25.5 (4.3)	*P* = 0.7
Mean post-op ICP (SD)	19 (3.3)	14.3 (1.6)	*P* < 0.00001
Mean post-op PbO2 (SD)	27.4 (2.6)	30.4 (3.3)	*P* < 0.00001

*DC* Decompressive craniectomy group; *AC* Adjuvant cisternostomy group; *SD* Standard deviation; *pts.* Patients; *ICP* Intracranial pressure; *PbO2* Brain tissue oxygen partial pressure

*Statistically significant

In the DC group, 11 patients (50%) required osmotherapy for refractory ICP compared with 3 patients (16.7%) in the AC group. Overall, bolus osmotherapy were administered at a mean of 2.8 (mannitol and/or hypertonic saline solution) per patient in the DC group compared with 0.2 in the AC group (Table 1) (*p* = 0.02).

**Fig. 4:**
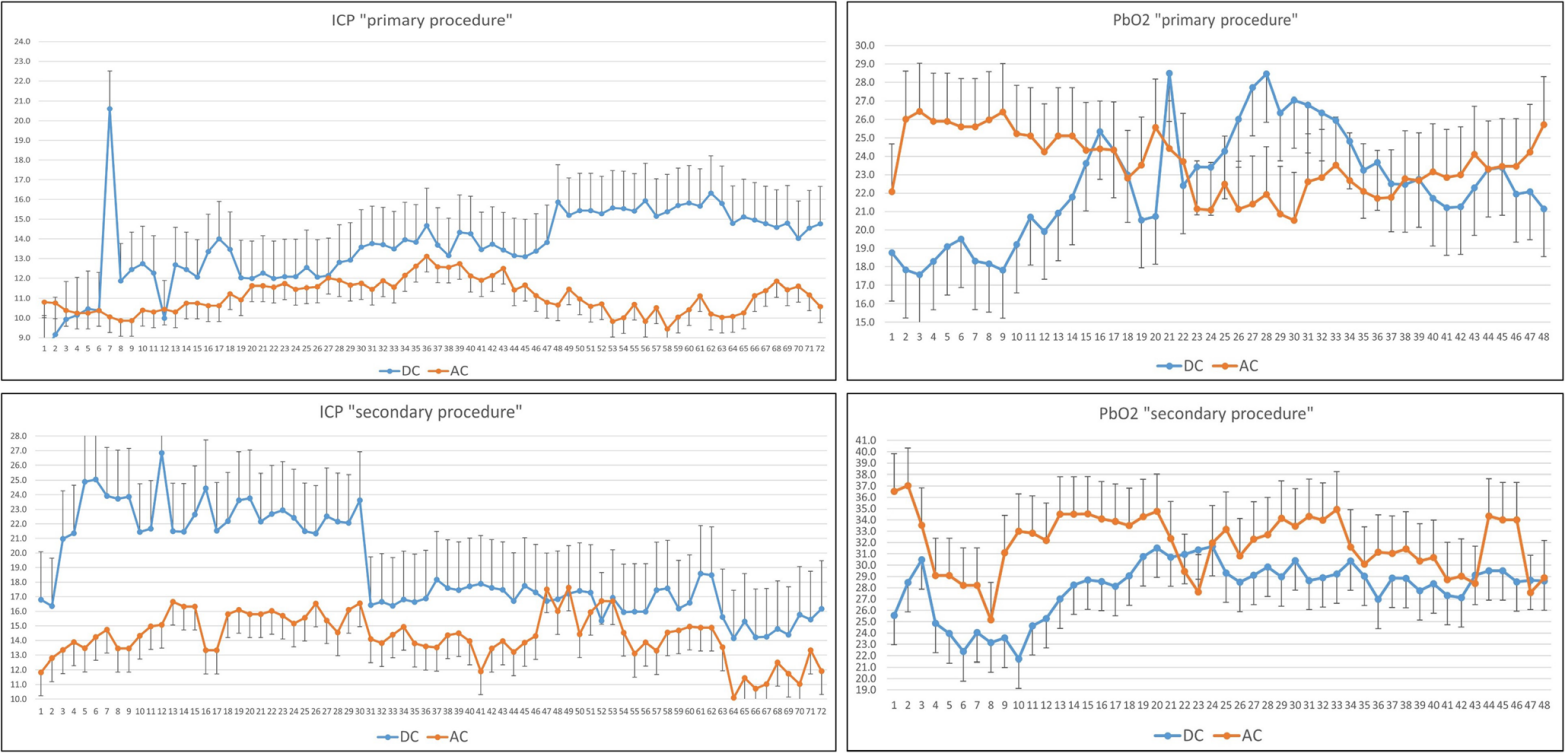
Neuromonitoring data (ICP and PbO2) showing the mean hourly values after surgery in the primary and secondary procedure subgroups

The subgroup analysis of neuromonitoring data also revealed significant differences between the two groups. When surgery was performed as a primary procedure, patients with AC had a significantly lower ICP in the 72 h after surgery (*p* = 0.0001) that correlated with a significant difference of PbO2 in the first 24 h (Table 4). In the secondary procedure subgroup analysis, patients with DC and AC had similar mean preoperative ICP and PbO2 values (*p* = 0.7). Patients with AC had an immediate normalization of ICP (*p* = 0.0001) and PbO2 values (*p* = 0.0001) (Table 4). This difference was more marked within the first 24 h while the DC group had ICP mean values above 20 mmHg (Fig. 4).

## Discussion

Overall, our retrospective analyses showed that, despite a significant higher percentage of unilateral mydriasis and globally worse radiological score, patients who underwent an adjuvant cisternostomy had a better clinical outcome (overall group and “primary procedure” subgroup), ICP control and brain tissue oxygenation when compared with a population of patients treated with DC alone, with more than 60% of patients presenting a favorable outcome. Moreover, our data also show that cisternostomy has a marked effect on the duration of ICU stay (mean 11.9 days in the AC vs 16.9 days in DC) and ventilation (mean 8.5 days in the AC vs. 12.9 days in the DC), parameters that certainly influence the clinical outcome of the patients.

When surgery is performed as a primary procedure, the addition of cisternostomy showed a clear improvement of the clinical outcome. In the group of patients who were treated with primary surgery, 8 of 13 patients with AC (61.5%) had a favorable outcome in comparison with 2 of 11 patients (18.2%) with DC. These groups were found to be homogenous with respect to clinical and radiological presentation (Tables 2 and 3, Fig. 2). This enhanced clinical outcome is also better than that found in published literature with respect to patients presenting with mass lesions who are candidates for urgent and early surgery [2, 21, 28]. Leitgeb et al. described a large series of patients who had surgery as a primary procedure for sTBI with an early mortality rate of 46.7% and only 32.2% of the patients experienced a favorable outcome. Moreover, the results in terms of ICP control in our cohort were remarkable, noting that primary DC has been associated with a persistent intracranial hypertension in a high percentage of patients, as described by the group of Servadei [29]. In their study, despite primary DC, a further interventional treatment was necessary in a high rate of patients (barbiturate coma 20.6%, external ventricular drainage 11.8%, and DC diameter widening 2.9%).

The second case scenario is a sTBI with a refractory ICP control despite maximal medical management. In our subgroup analysis, though we failed to show a significant better outcome, the cisternostomy procedure led to a clear and immediate normalization of ICP and PbO2 without the need to give osmotherapy following surgery. Moreover, patients in the AC subgroup had mean ICP values always below 15 mmHg, a threshold that has proved to be a positive prognostic factor in case of sTBI [31]. This subgroup also differs in terms of PbO2 values; patients with AC showed higher mean values since the immediate postoperative course, and this was more important within the first 24 h. These results reflect an improved brain oxygenation, which was shown to have a positive impact on outcome [24, 34]. Despite these encouraging neuromonitoring results, the addition of cisternostomy to DC did not affect the clinical outcome in case of a secondary procedure. The low number of patients could in part explain these findings, but also, we may infer that in this specific subgroup, the surgical procedure was performed after failure of intensive medical management and when the secondary brain damage was already ongoing (surgery performed at a mean of 4 days following trauma). In the light of the immediate normalization of the neuromonitoring data in the AC group, we should consider if earlier surgical management could prevent this secondary brain damage and improve clinical outcomes.

The “standard” technique of opening the cisterns as commonly done in surgeries for ruptured aneurysms or skull base tumors is known to have a significant and immediate impact on brain swelling, thereby enabling a lax brain during these surgeries. This positive effect can further be enhanced in the postoperative phase by the placement of a cisternal drain, which is therefore conceptually different from EVD [12].

Contrary to the earlier concepts of CSF circulation, we now know that CSF is continuously produced and absorbed in the entire CSF system with a major role played by the paravascular Virchow-Robin spaces [5, 25, 26]. Illiff and colleagues, in particular, demonstrated that the fluid exchange between cisterns and brain parenchyma is significantly more intense than exchange seen between the ventricles and brain parenchyma. The authors found that such perivascular CSF circulation was mediated by the astroglial water channel aquaporin-4, thus leading to the term “glymphatic” system [18]. The same authors also demonstrated that the function of the glymphatic system following TBI is impaired with a subsequent decrease in the drainage of interstitial fluid [19]. In the setting of severe TBI, there is almost always a significant amount of associated subarachnoid hemorrhage. It could be hypothesized that traumatic SAH could make the pressure rise within the cisternal compartment (by clogging the natural CSF drainage pathways), thus producing an outflow congestion or shift of fluid towards the brain [15] which then leads to brain swelling via the development of “CSF shift edema” [8, 9, 12]. By opening the basal cisterns and lamina terminalis, cisternostomy enables the catheter to drain both the cisternal and ventricular compartments, and this may therefore reverse this shift of fluid to the intraparenchymal compartment, thus alleviating brain swelling.

Cisternostomy offers the possibility to drain a significantly larger quantity of CSF, a mean of 207 ml/day in our series, which is remarkable in the setting of severe TBI (when compared with the traditional drainage through an EVD). In severe TBI, the ventricular system is often collapsed and the amount of CSF that can be obtained by a standard EVD often does not allow to efficiently control ICP over time [35]. Moreover, the debris and blood clots within a small ventricular cavity frequently lead to EVD failure. In this series, we had no instances of failure of cisternal drainage. This could also be related to the fact that the cisternostomy procedure, in itself, allows an extensive washout of all blood clots and brain debris from the cisterns, thus reducing the probability of failure.

The eventual outcome of TBI patients depends on the primary insult and on the secondary injury (the latest including intracranial hypertension and cerebral hypoperfusion). Despite its importance, optimization of ICP is not sufficient, in itself, to improve outcome because there is an important role for metabolic, excitotoxic, and inflammatory factors that contribute to the secondary brain injury. The adjuvant cisternostomy, by improving the brain oxygenation may also affect the clinical outcome, as has been shown in previous study [24, 34].

Experience from trauma surgery shows that durotomy in the context of sTBI can sometimes result in massive intraoperative brain swelling/herniation with catastrophic consequences [1]. The surgical technique of cisternostomy begins with a small basal dural opening (after epidural bony decompression), and this enables a quick access to the basal cisterns, resulting in achieving a lax brain, quite early in the procedure. The durotomy over the rest of convexity surface is then completed in a second phase, thus preventing the kinking of cortical veins, lacerations of cerebral cortex on the bone edge, and the potential damage added by cerebral swelling [1, 20, 32].

Our study showed that in the AC group there was a significant trend towards less brain deformation (calculated in terms of outward brain herniation) and also allowing an earlier cranioplasty procedure. This interesting finding implies that cisternostomy may counteract the negative effects of DC and affect the long-term outcome [30].

### Limits of the study

This study has limitations related to its retrospective observational nature, which is associated with several methodological drawbacks common to retrospective data. Due to the limited number of patients, the results of comparative analysis of the subgroups should be taken with caution, and further clinical studies with larger patient cohorts are needed to confirm these findings. The surgeries in this series were performed by multiple surgeons over the years. While all surgeons were board certified and experienced in trauma surgery, the AC group patients were operated on by surgeons with an added expertise in acute aneurysm surgery. The surgical procedure of a cisternostomy requires the necessary instrumentation and skills in skull base and vascular surgery. A universal application of this technique at trauma care centers could be problematic. The favorable results of this surgical series cannot be used to justify attempts at cisternal access in tight brains without adequate training [11, 13]. The implementation of this technique is easy in centers that perform surgery for aSAH on a regular basis. Centers that have high volume elective skull base surgery programs will also be able to quickly implement this technique, as part of trauma surgery protocols.

## Conclusion

The surgical procedure of an adjuvant cisternostomy is safe and feasible in the context of traumatic brain injury. Our preliminary single-center data indicate a clinically relevant improvement in patient clinical outcome (both at early and long term). Continued cisternal drainage also improved brain oxygen and enabled a better control of ICP, thereby lessening the need for osmotherapy in the postoperative period. Additional benefits included reduced duration of mechanical ventilation and ICU stay, and allowing an earlier cranioplasty. These promising results need further confirmation by larger clinical studies through multi-institutional efforts. Further subgroup analysis should then be able to elucidate the ideal timing for this procedure and help predict which patients will mostly benefit from this novel surgical therapeutic approach.
